# Supplementary material for: Complexome profiling of the Chlamydomonas psb28 mutant reveals TEF5 as an early PSII assembly factor
Source: Plant Cell. 2025 Mar 17;37(6):koaf055. doi: 10.1093/plcell/koaf055 (PMC12164590; doi:10.1093/plcell/koaf055)
Supplement: koaf055_Supplementary_Data [file koaf055_supplementary_data.zip › psb28_interactive.zip!/plots/intensity/qualityPlots/peptides/CPLD59_Cre09.g404000.t1.1.html]

### Cre09.g404000.t1.1

Trivial name: CPLD59  
SeqCoverage [median]: 19.10  
Mass: 22.61 kDa  
  
 

| Sequence | MissedCleavage | Mass [Da] | wtIntensity | muIntensity | Score |
| --- | --- | --- | --- | --- | --- |
| ANFEGADMTNAVVDR | 0 | 1608.72 | 0.00 | 468.84 | 120.53 |
| ANFEGADMTNAVVDRVDFTNANLK | 1 | 2611.23 | 0.00 | 1193.25 | 42.71 |
| ANFEGADMTNAVVDRVDFTNANLKR | 2 | 2767.33 | 0.00 | 11425.85 | 127.28 |
| GYVGNTIGQANAVSDK | 0 | 1592.78 | 0.00 | 1878.43 | 191.48 |
| VDFTNANLKR | 1 | 1176.63 | 372.83 | 1738.77 | 46.16 |
|  |  |  | 372.8 | 16705.1 |  |
